# Formation and Characterization of Bifunctional Nanoparticles Fabricated from Insoluble Rice Peptide Aggregate: Effect of Enzymes

**DOI:** 10.3390/foods14223974

**Published:** 2025-11-20

**Authors:** Xinxia Zhang, Shengze Ma, Ting Li, Li Wang

**Affiliations:** 1 Key Laboratory of Carbohydrate Chemistry and Biotechnology, Ministry of Education, Jiangnan University, Lihu Road 1800, Wuxi 214122, China; 8202108006@jiangnan.edu.cn (X.Z.);; 2 National Engineering Research Center for Cereal Fermentation and Food Biomanufacturing, Jiangnan University, Lihu Road 1800, Wuxi 214122, China; 3 Jiangsu Provincial Engineering Research Center for Bioactive Product Processing, Jiangnan University, Lihu Road 1800, Wuxi 214122, China; 4 School of Food Science and Technology, Jiangnan University, Lihu Road 1800, Wuxi 214122, China; 5 Collaborative Innovation Center of food Safety and Quality Control in Jiangsu Province, Jiangnan University, Lihu Road 1800, Wuxi 214122, China

**Keywords:** rice peptide aggregate, bifunctional nanoparticles, high internal phase emulsions

## Abstract

This study systematically investigates the effects of enzyme type (Alcalase, Trypsin, Protamex) on the properties of rice peptide nanoparticles (RPNs) and their efficacy in stabilizing high internal phase emulsions (HIPEs). RPNs prepared with Alcalase (RPNs-alc) exhibited the smallest particle size (≈379.6 nm), a uniform unimodal distribution, the highest content of hydrophobic amino acid, and the strongest DPPH (2,2-Diphenyl-1-picrylhydrazyl) radical scavenging activity (57.32%). In contrast, RPNs from Protamex (RPNs-pro) showed larger, heterogeneous particles with a bimodal distribution and lower antioxidant capacity. Interfacial characterization revealed that RPNs-alc had a superior three-phase contact angle, indicating enhanced interfacial activity. Structural stability analysis confirmed that hydrophobic interactions and hydrogen bonds are the primary forces maintaining all RPNs. Consequently, HIPEs stabilized by RPNs-alc and RPNs-typ displayed solid-like behavior and a regular network microstructure, leading to exceptional physical stability. Conversely, RPNs-pro led to unstable HIPEs with non-uniform droplets and interfacial aggregation, promoting droplet flocculation. These findings demonstrate that enzyme selection critically determines the functional properties of RPNs, with Alcalase-derived RPNs being the most effective bifunctional particles, offering a viable pathway for valorizing proteolytic by-products in fabricating stable, antioxidant-rich Pickering emulsions.

## 1. Introduction

Rice protein, an important cereal protein, has attracted extensive attention because of its rational amino acid composition, rich nutritional value, digestibility, and hypoallergenic properties [1,2]. Recently, enzymatic hydrolysis has been widely applied to prepare rice protein-based bioactive peptides which satisfy the demand for improving human wellness through daily diet [3,4,5,6]. Although enzymatic hydrolysis significantly decreases the hydrophobic property of rice protein and broadens its application in potential food nutraceuticals, undesirable insoluble rice peptide aggregates (IRPAs, up to 30% of rice protein) [7] form during the hydrolysis process because of the strong hydrophobicity and compact structure of rice protein. Moreover, different protein enzymes are used in order to obtain peptides with special bioactivity (such as Alcalase-based antioxidant peptides [8] and Trypsin-based ACE inhibitory peptides [9]), thus resulting in great differences in the properties of the IRPAs. However, few scholars have explored the properties and application of insoluble aggregates produced by various enzymes. These large IRPAs are normally used as low-cost animal feed or discarded, ignoring their bioactive and nutritional potential, which hinder their sustainable use in the food industry.

In recent years, biopolymer nanostructures, based on protein or polypeptides, have attracted great attention from food researchers due to their potential in functional food, pharmaceutical, and cosmetic fields [10,11,12,13,14]. Amphiphilic peptides have been widely used to construct nanostructures, such as self-assembled egg yolk peptide micellar nanoparticles [15] and sono-assembled soy peptide nanoparticles [16]. These peptide-based nanostructures, which have good biocompatibility, are suitable for fabricating delivery vehicles for various active compounds (such as curcumin [17]). On the other hand, these peptide-based nanostructures have both hydrophilic and hydrophobic groups and can potentially be used as emulsifiers and stabilizers for Pickering emulsions [18]. Moreover, peptide-based nanostructures possess inherent antioxidant activity that can confer excellent antioxidant stability to emulsions. In this case, peptide-based nanostructures have great potential in the preparation of high-quality emulsions. However, reports on this aspect are very limited in the field of food processing.

As mentioned above, the IRPAs formed upon proteolysis are generally wasted. Our previous study demonstrated that ultrasonic treatment could successfully induce IRPAs self-assembly into a new type of rice peptide nanoparticles (RPNs) with good antioxidant activity. Moreover, the RPNs were further shown to be able to act as excellent bifunctional interfacial stabilizers that can endow emulsions both physical stability and antioxidant stability [7]. This investigation provides a new idea for the application of the insoluble aggregates produced during hydrolysis. However, few studies could be found in this field. Zhang et al. [19] found that this type of enzyme has been proven to exert a great influence on the structural and functional properties of the insoluble aggregates because of the different active site of different enzymes. This may further influence the biological activity and interface stability of the resultant nanoparticles. However, there is no relevant research at present.

Therefore, we investigate how enzyme type influences the properties of the RPNs. Three types of widely used enzymes including Alcalase, Trypsin and Protamex, which can cleave different peptide bonds, were selected to produce IPRAs. Ultrasound was used to induce IRPA assembly to form RPNs. The physicochemical properties (particle size, morphology, structural properties, antioxidant activity) were firstly analyzed. The interfacial properties were then determined. In addition, the RPNs were used to fabricate high internal phase emulsions (HIPEs). We expect that the obtained results could open up a new research area on the application of proteolytic by-products.

## 2. Materials and Methods

### 2.1. Materials and Chemicals

Rice protein was provided by Shanyuan Biotechnology Co., Ltd. (Wuxi, China). Alcalase (2.4 L), Trypsin (B), and Protamex were purchased from Novozymes (Beijing, China). DPPH and ferrozine were obtained from Sigma (St Louis, MO, USA). All chemicals and reagents were of analytical grade or higher.

### 2.2. Preparation of Insoluble Rice Peptide Aggregates (IRPAs)

The hydrolysis followed the conditions given in the following parentheses: Alcalase 2.4 L (pH 8.5, 55 °C), Trypsin (pH 8, 50 °C), and Protamex (pH 7, 50 °C).

The rice protein suspension (5% *w*/*v*) was stirred for 30 min at the specific optimum temperature for each protease. Enzymatic hydrolysis was then initiated at an enzyme/substrate ratio of 1:100 (*w*/*w*) and allowed to proceed for 2 h under optimal conditions, with the pH being continuously regulated using 1 M NaOH. To inactivate the enzymes, the mixtures were heated in a boiling water bath for 10 min. After cooling and pH adjustment to 7.0, the hydrolysates were centrifuged at 10,000× *g* for 20 min. The precipitate was collected, washed three times with distilled water, and subsequently lyophilized.

### 2.3. Rice Peptide Nanoparticles (RPNs) Preparation

A 3% (*w*/*v*) IRPA dispersion was prepared using deionized water. The dispersion was first hydrated by stirring for 2 h, followed by sonication using an ultrasonic processor (FS-1200N, SHSONIC Instrument Co., Ltd., Shanghai, China) equipped with a 10 mm probe (360 W, 20 kHz) for 15 min. The resulting RPN dispersion was then obtained by centrifuging the sonicated mixture at 10,000× *g* for 10 min.

### 2.4. Particle Size and Zeta Potential

The particle size and zeta potential of the RPNs, diluted to 0.05% (*w*/*v*) using deionized water, were determined using a Zetasizer Nano (Malvern Instruments Ltd., Malvern, UK), following a previously established method [7].

### 2.5. Scanning Electron Microscopy (SEM)

A 5 μL aliquot of the RPNs dispersion was deposited on a monocrystalline silicon substrate and air-dried. The prepared sample was then gold-sputtered under vacuum using an ion coater and imaged with a cold field emission scanning electron microscope (SU8100, Hitachi High-Technologies, Tokyo, Japan) at 10,000× magnification.

### 2.6. Transmission Electron Microscope (TEM)

A 10 μL aliquot of the diluted RPN dispersion (0.1 wt%) was deposited onto a carbon-coated copper grid. After 60 s, the excess liquid was blotted with filter paper. The grid was subsequently negatively stained with 10 μL of 1% phosphotungstic acid for 60 s. Micrographs were acquired using a JEM-2100F transmission electron microscope (JEOL, Tokyo, Japan) operating at 100 kV with a magnification of 70,000×.

### 2.7. Contact Angle

The three-phase contact angle of the RPNs was determined to assess their wettability, employing an OCA 20 AMP apparatus (Dataphysics Instruments, Filderstadt, Germany) via the sessile drop method. Powdered RPN pellets were prepared and immersed in peanut oil within an optical glass cuvette. A 20 μL droplet of distilled water was then carefully dispensed onto the pellet surface using a high-precision syringe and allowed to equilibrate for 3 min. The contact angle was automatically calculated by the instrument’s software, applying a Young–Laplace fit to the captured droplet image.

### 2.8. Inner Interactive Forces of RPNs

To probe the intra-particle forces stabilizing the RPNs, we monitored changes in the particle size of ultrasonicated dispersions upon treatment with various chemical disruptors. These included distilled water (control), 0.5% sodium lauryl sulfate (SDS), 6 M urea, and 30 mM dithiothreitol (DTT), both individually and in combination. After 30 min of incubation, the particle size was measured using dynamic light scattering (DLS), as detailed in Section 2.4.

### 2.9. Amino Acid Analysis

Next, 200 mg RPHs underwent hydrolysis in sealed, evacuated glass tubes with 6 M HCl at 110 °C for 24 h. Following evaporation under nitrogen at 60 °C, the hydrolysates were brought to a volume of 100 mL with water and filtered. Amino acid analysis was carried out on the filtrate using an automated amino acid analyzer (L-8800, Hitachi, Tokyo, Japan).

### 2.10. DPPH Radical Scavenging Activity

All RPN dispersions were diluted to 0.5% (*w*/*v*). A 100 μL aliquot of the diluted dispersion was mixed with an equal volume of 0.25 mM DPPH (in ethanol), followed by incubation in the dark at 25 °C for 30 min. The absorbance at 517 nm was measured using a microplate reader (SH-1000, Hitachi, Tokyo, Japan). The DPPH scavenging activity was calculated as

(1) Scavenging Activity (%) = 100 − [(A_s1_ − A_s2_)/A_c_] × 100

where A_c_, A_s1_, and A_s2_ represent the absorbance of the control, sample, and sample background (without DPPH), respectively.

### 2.11. Iron (Fe^2+^) Chelating Activity

The Fe^2+^ chelating activity was evaluated as follows: RPN dispersions were first diluted to 0.5%. A 100 μL aliquot of the diluted sample was mixed with 150 μL of distilled water, followed by the addition of 10 μL of 2 mM ferrous chloride. After a 3 min reaction, the mixture was inhibited by adding 20 μL of 5 mM ferrozine solution. The final mixture was then vigorously vortexed and allowed to stand at room temperature for 10 min before measuring the absorbance at 562 nm using a microplate reader (SH-1000, Hitachi, Japan). The chelating activity was calculated as

(2) Fe^2+^ Chelating Activity (%) = 100 − [(A_s1_ − A_s2_)/A_c_] × 100

where A_c_, A_s1_, and A_s2_ represent the absorbance of the control, sample, and sample background (without Fe^2+^), respectively.

### 2.12. Preparation of High Internal Phase Emulsion (HIPE)

An HIPE was prepared by homogenizing a 4% (*w*/*v*) RPN dispersion with an oil phase (φ = 0.80) at 15,000 rpm for 2 min using an IKA T18BS25 homogenizer.

### 2.13. Confocal Laser Scanning Microscopy (CLSM)

The HIPE sample (1 mL) was first stained with 50 μL of a 1% Nile Blue solution (prepared in isopropyl alcohol containing 1% distilled water). Subsequently, a 10 μL aliquot of the stained HIPE was placed on a microscope slide, covered with a coverslip, and imaged using an LSM880 confocal laser scanning microscope (Zeiss, Oberkochen, Germany). Furthermore, the sample was observed using a 40× magnification lens with the Argon–Krypton and Helium–Neon laser excitation wavelengths at 488 nm and 633 nm for protein and oil, respectively.

### 2.14. Statistical Analysis

All experiments were performed in triplicate, and data are expressed as the mean values. Statistical significance (*p* < 0.05) was determined using SPSS 18.0 software.

## 3. Results

### 3.1. Yield of the Insoluble Rice Peptide Aggregates (IRPAs)

**Figure 1:**
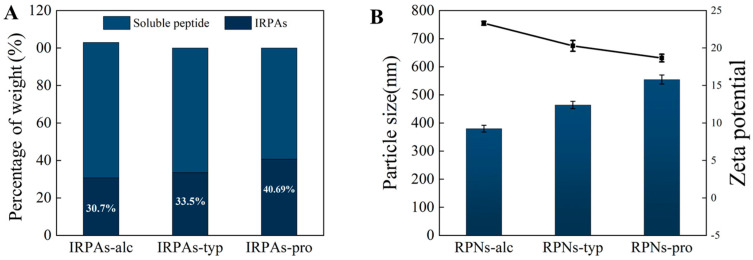
(**A**) Yield of IRPAs produced by various enzymes. (**B**) Effect of enzyme type on particle size and zeta potential of the resultant RPNs. Black line represents variation in zeta potential.

Various peptide chains with hydrophobic amino acid residues will be released during protein hydrolysis. These hydrophobic residues tend to aggregate through peptide–peptide/protein interactions and further form insoluble peptide aggregates [20]. Moreover, these aggregates additionally incorporate a component formed through heat-induced protein aggregation. The production of peptide aggregates differs according to the enzymes used during protein hydrolysis and the results are shown in Figure 1. The percentages of IRPAs produced by hydrolysis were 30.7 ± 0.31% (Alcalase), 33.5 ± 0.27% (Trypsin), and 40.69 ± 0.38% (Protamex). This difference is mainly due to the different degrees of hydrolysis of different enzymes at the same time.

### 3.2. Size and Morphological Properties of RPNs

**Figure 2:**
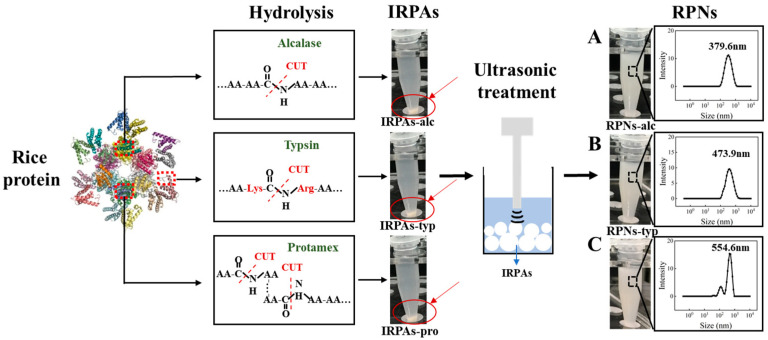
The visual observation and particle size distributions of IRPA dispersion and the resultant RPN dispersion obtained from Alcalase (**A**), Trypsin (**B**), Protamex (**C**).

Compared with the control IRPA dispersion with marked precipitate, it can be observed that IRPAs obtained from different enzymes transformed into uniform dispersions under ultrasonic induction (visual observation in Figure 2). This phenomenon could be attributed to the breaking up of non-covalent interactions induced by ultrasonication which converted large aggregates into smaller ones [21]. However, there are big differences in the particle size of the resultant RPN dispersion (Figure 1B). RPN-alc (RPNs obtained from IRPAs produced with Alcalase) displayed the minimum particle size around 379.6 nm, while RPNs-typ (RPNs obtained from IRPAs produced with Trypsin) and RPNs-pro (RPNs obtained from IRPAs produced with Protamex) present bigger particle sizes. On the other hand, RPNs-alc and RPNs-typ displayed unimodal size distributions (as shown in Figure 2A,B) when compared to RPNs-pro (RPNs obtained from IRPAs produced with Protamex) with a bimodal distribution (Figure 2C), suggesting uniform size distributions of the resultant RPNs. The difference in particle size is closely related to the property of the enzyme itself. Alcalase can undifferentially hydrolyze the peptide bonds of the protein (both internal and external) and chop the protein into smaller fragments [22], thus resulting in aggregates containing more small molecular fragments, resulting in smaller particles under ultrasonic action. Trypsin is a specific endopeptidase that selectively severs peptide bonds formed at the carboxyl ends of lysine and arginine residues [23], producing bigger aggregates than those produced from Alcalase. In the case of a complex protease, it can either cut off the end of the peptide chain to release amino acids or cut the peptide bond from the inside of the peptide chain to produce large peptides, so it produces both small and large molecular fragments (Figure 2C). Moreover, RPNs-alc and RPNs-typ have negative zeta potentials of −20.29 ± 0.73 and −23.32 ± 0.26 (Figure 1B), respectively, which were sufficient to maintain the colloidal stability of RPNs by electrostatic repulsion in water [24,25].

**Figure 3:**
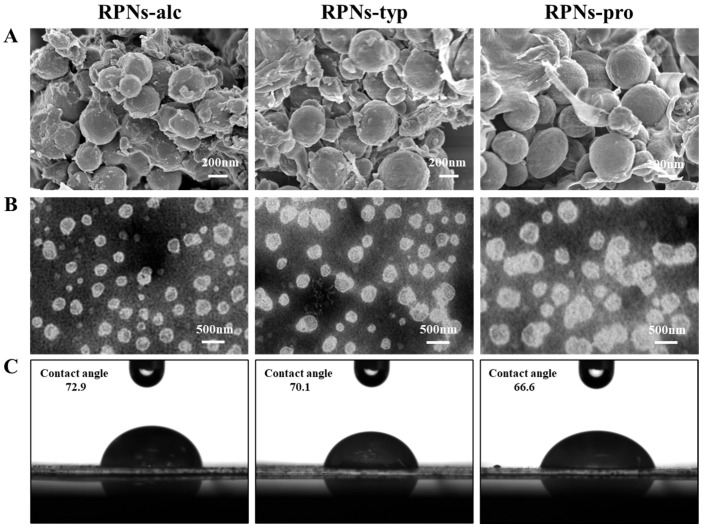
(**A**) SEM images, (**B**) TEM images, and (**C**) contact angles of RPNs obtained from Alcalase, Trypsin, and Protamex.

To comprehensively characterize the structural attributes of the rice peptide nanoparticles (RPNs) derived from different enzymatic sources, scanning electron microscopy (SEM) and transmission electron microscopy (TEM) were employed to examine the morphologies of the freshly prepared nanoparticles, as shown in Figure 3A,B. The electron micrographs revealed that all three types of RPNs consistently exhibited spherical shapes. This consistent morphology indicates that the general formation mechanism of nanoparticles from insoluble rice peptide aggregates (IRPAs) under ultrasonication is not fundamentally altered by the choice of protease, and that the spherical form is an intrinsic characteristic of the self-assembled RPNs under the given preparation conditions. However, significant differences were observed in their size distribution and state of aggregation, which were critically influenced by the enzymatic origin. RPNs-alc and RPNs-typ displayed a uniform particle distribution with excellent dispersion, characterized by well-separated, discrete spherical nanoparticles. This homogeneity facilitates dense and orderly packing at interfaces, which is a key determinant of their superior emulsifying performance. In stark contrast, the RPNs-pro sample exhibited substantial heterogeneity in particle size and a clear tendency to form large, irregular aggregates, where multiple particles clustered together. This aggregated state compromises their ability to form a continuous and cohesive interfacial film.

The morphological features observed via SEM and TEM are in direct agreement with the particle size distribution data obtained from dynamic light scattering (Figure 2). The unimodal, narrow size distributions of RPNs-alc and RPNs-typ correlate with their uniform appearance under microscopy, while the broader, bimodal distribution and larger mean size of RPNs-pro directly correspond to the observed size variability and aggregation. This consistency across analytical techniques confirms that the enzyme-specific hydrolysis patterns ultimately dictate the nano-scale assembly of the peptides, which in turn governs the macroscopic functionality of the RPNs as emulsifiers.

### 3.3. Three-Phase Contact Angle of RPNs

The three-phase contact angle formed between oil and water interfaces serves as a key indicator of the interfacial wettability of particles, providing critical insight into their adsorption behavior at liquid–liquid interfaces. Furthermore, this wettability directly correlates with the capacity of particles to stabilize Pickering emulsions. Thus, the three-phase contact angle of RPNs-alc, RPNs-typ, and RPNs-pro were detected to compare their interfacial properties (Figure 3C). The results clearly demonstrated that enzyme selection significantly affected the interfacial wettability of the resulting RPNs. Specifically, RPNs-alc exhibited the largest contact angle, indicating its superior adsorption capability at the oil–water interface compared with RPNs-typ and RPNs-pro. This enhanced interfacial activity can be attributed to the unique surface composition and structure of RPNs-alc, which facilitates more effective particle adsorption and the formation of a stronger mechanical barrier at the interface.

### 3.4. Intra-Particle Interactive Forces of RPNs

**Figure 4:**
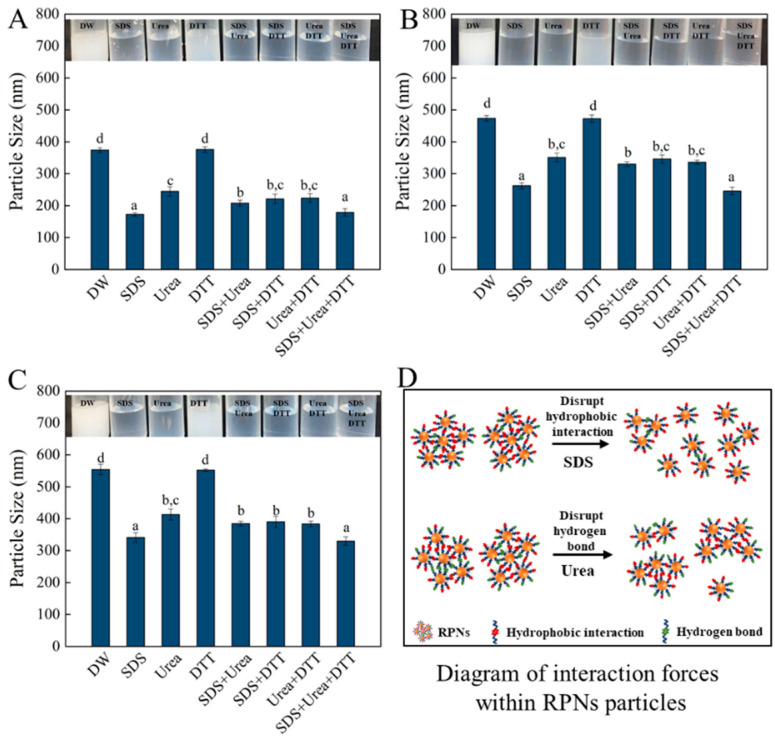
The effect of different protein denaturant solvents on the visual appearance (**upper panels**) and particle size (**lower panels**) of RPNs obtained from Alcalase (**A**), Trypsin (**B**), and Protamex (**C**). Diagram of interaction forces within RPNs (**D**). The lowercase letters indicate a significant difference (*p* < 0.05).

To illustrate the intermolecular force that maintains the structure of the nanoparticles obtained from different enzymes, the appearance and particle size changes in freshly prepared RPNs under the action of different protein disruptors (urea, SDS, DTT) are measured, and the results were shown in Figure 4. Urea, SDS, and DTT are commonly used to break hydrogen bonds, hydrophobic interactions, and disulfide bonds, respectively [26]. As can be seen from the figure, the trend in the changes of appearance of the three kinds of nanoparticles prepared by different enzymes is the same. When DTT was alone, the appearance of the particles did not change significantly. However, when SDS and urea were used alone or in combination, the particle dispersion solution became clearer (especially SDS), which indicated that hydrophobic interactions and hydrogen bonds were the main forces that maintained the particle structure. In terms of particle size variation, the variation trend of particle size of the three nanoparticles is consistent regardless of their size. The particle sizes of the RPNs were significantly decreased when 0.5% SDS and 6 M urea were present alone or in combination, while the particles size of the RPNs were not significantly changed when 30 mM DTT was present alone, which further demonstrated that hydrophobic interactions and hydrogen bonds were the main forces maintaining the structure of the RPNs. These results also indicate that although the sizes of the nanoparticles prepared by different enzymes were different, the main force maintaining the structure of the nanoparticles was the same.

### 3.5. Amino Acid Composition of RPNs

The effects of enzyme type on the amino acid (AA) composition of RPNs are summarized in Table 1. The contents of hydrophobic amino acids in the three kinds of RPNs were significantly higher than that in RP, indicating that hydrophobic amino acids tend to be concentrated in IRPAs during enzymatic hydrolysis, resulting in the stronger hydrophobicity of RPNs than RP. In addition, the type of enzyme had a significant effect on the amino acid composition of RPNs. There were significant differences in the hydrophobic amino acids content of RPNs obtained with various proteases. In decreasing order they were RPNs-alc > RPNs-typ > RPNs-pro. Studies have shown that hydrophobic peptides are generally wrapped by hydrophilic peptides, so hydrophilic peptides are hydrolyzed first in the enzymatic hydrolysis process and mostly exist in soluble protein peptides, while hydrophobic peptides aggregate into insoluble aggregates through hydrophobicity [27]. Alcalase has the highest hydrolysis intensity [8], resulting in smaller peptides and more hydrophobic amino acids exposed, which increases the hydrophobic aggregation between peptides and leads to more hydrophobic amino acids in RPNS-alc.

Notably, the essential amino acid (EAA) content in RPNs (41.37–42.65 g/100 g) was substantially higher than that in RP (36.55 g/100 g). This difference can be attributed to the fact that most EAAs are also hydrophobic, a group which was more abundant in RPNs. 

### 3.6. Antioxidant Activity of RPNs

**Figure 5:**
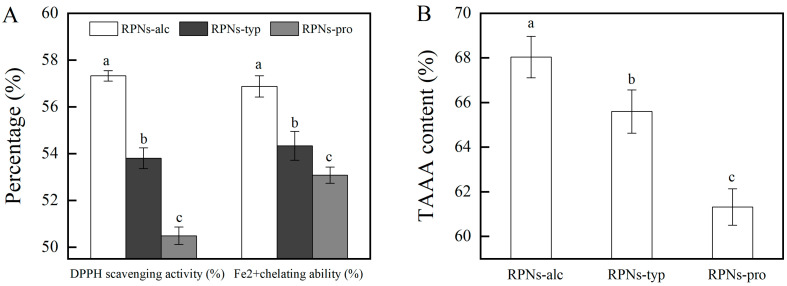
(**A**) DPPH scavenging activity and Fe^2+^ chelating activity of RPNs obtained from different enzymes. (**B**) TAAA (total antioxidant related amino acid) content in RPNs different enzymes. The lowercase letters indicate a significant difference (*p* < 0.05).

It is known that an emulsion system rich in oil is easily oxidized by free radicals and ions in the emulsion, resulting in food flavor deterioration and nutritional loss. Chemical antioxidants can effectively inhibit oil oxidation. However, their use has been limited because of the demand of natural foods from consumers. Therefore, a bifunctional emulsifier with both interface stability and oxidation resistance has a unique advantage in fabricating food emulsions. Our previous study proved that RPNs obtained from Alcalase could effectively inhabit oil oxidation and fabricate emulsions with good physical stability and oxidative stability [7]. In this study, the antioxidant activity of RPNs obtained from different enzymes were compared using the DPPH and Fe^2+^ chelating methods. As shown in Figure 5A, the DPPH scavenging rate of RPNs obtained from different enzymes ranged from 50.48% to 57.32%. All three kinds of RPNs present a greater free radical clearing ability compared with the corresponding IRPAs (DPPH scavenging rate ranged from 15.91% to 17.23%). It should be noted that the enzyme type had a significant effect on the DPPH scavenging activity of RPNs. RPNs-alc present the best DPPH radical scavenging capacity, with the highest value of 57.32 ± 0.22%. The DPPH radical scavenging rate of RPNs-typ and RPNs-pro was 6.14% and 11.92% lower than that of RPNs-alc, respectively. A similar trend was also observed for the Fe^2+^ scavenging ability of several RPNs. Several factors contribute to this phenomenon. On the one hand, there are differences in the action sites of enzymes and different peptides will be released during hydrolysis, resulting in different amino acid compositions [28] of IRPAs obtained from proteins hydrolyzed by different enzymes. As can be seen in Figure 5B, the content of antioxidant-related amino acids in RPNs-alc was as high as 68.04 ± 0.93%, which was 3.59% and 9.88% higher than that in RPNs-typ and RPNs-pro, respectively. This means that there are more action sites in RPNs-alc that can capture free radicals, thus showing a stronger antioxidant capacity. On the other hand, RPNs-alc showed the smallest particle size among the three particles and the best dispersion in water, thus increasing the area of binding with free radicals [29] and enhancing the oxidation resistance. The results showed that RPNs prepared by different enzymes had some differences in antioxidant activity, but their antioxidant activity was much higher than IRPAs, indicating that they had the potential to become bifocal particles with both antioxidant activity and interface stability.

### 3.7. Characterization of HIPEs Stabilized by RPNs

**Figure 6:**
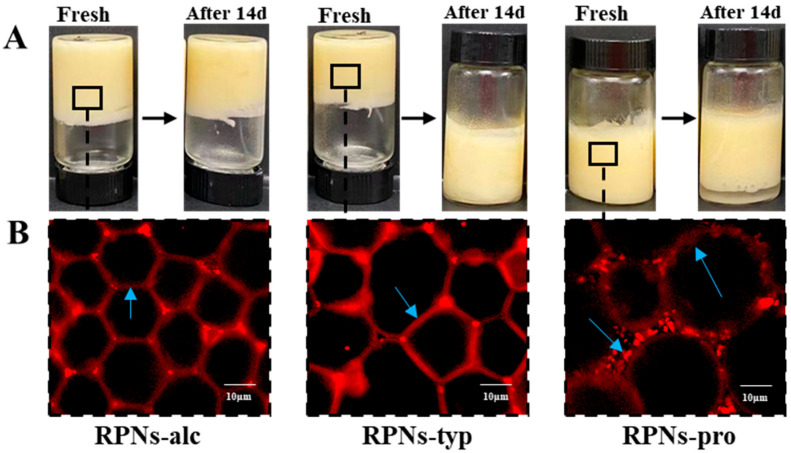
(**A**) Freshly prepared emulsion and emulsion stored for 14 days made by RPNs obtained from Alcalase, Trypsin, and Protamex. (**B**) CLSM images of fresh emulsion made by RPNs obtained from Alcalase, Trypsin, and Protamex.

As illustrated in Figure 6A, high internal phase emulsions (HIPEs) stabilized by RPNs from different enzymatic sources exhibited distinct macroscopic properties. As can be seen, all RPN types (RPNs-alc, RPNs-typ, and RPNs-pro) successfully formed HIPEs with an oil phase fraction (φ) of 0.80, demonstrating their general capability as Pickering stabilizers. However, marked differences were observed in their rheological behavior. HIPEs stabilized by RPNs-alc and RPNs-typ displayed solid-like elastic characteristics, maintaining their structural integrity without collapse or flow even when subjected to vial inversion. This indicates the formation of a strong, self-supporting network within the emulsion. In contrast, HIPEs prepared with RPNs-pro showed a more liquid-like behavior and failed to retain their shape upon inversion, suggesting weaker internal cohesion and inadequate stabilization.

Over a 14-day storage period, the stability of the HIPEs further highlighted the impact of enzyme selection. HIPEs stabilized by RPNs-alc showed no significant oil leakage or change in texture, maintaining their original height and homogeneity during storage. The HIPEs stabilized by RPNs-typ demonstrated increased fluidity and structural instability, as they could not maintain their integrity when the vial was inverted. In contrast, HIPEs stabilized by RPNs-pro exhibited distinct solid–liquid phase separation after 14 days of storage.

The macroscopic stability of the HIPEs directly correlated with their underlying microstructural architectures, as revealed in Figure 6B. HIPEs stabilized by RPNs-alc exhibited a highly ordered and continuous network structure, where uniformly sized oil droplets were tightly packed and separated by thin, interconnected particle-based films. This regular microstructure can be attributed to the small and monodisperse particle size of RPNs-alc (≈379.6 nm, as shown in Figure 2A), which facilitates rapid and irreversible adsorption at the oil–water interface during homogenization. The resulting efficient interfacial coverage forms a dense, coherent physical barrier. Furthermore, the optimal wettability of RPNs-alc ensured long-term emulsion stability by securing irreversible particle adsorption and offering effective anti-coalescence protection. In stark contrast, the HIPEs stabilized by RPNs-pro exhibited a non-uniform droplet size distribution and visible aggregates at the interface. These interfacial aggregates likely served as bridging points between adjacent oil droplets, enhancing droplet-droplet attraction through hydrophobic interactions. This phenomenon promoted droplet flocculation and ultimately led to macroscopic phase separation. The irregular microstructure of the RPNs-pro-based HIPEs reflects the polydisperse nature and poor interfacial packing capability of the stabilizer, which can be attributed to the large particle size and broad size distribution of RPNs-pro.

## 4. Conclusions

This study systematically investigated the effects of enzyme type (Alcalase, Trypsin, Protamex) on the properties of RPNs and their efficacy in stabilizing HIPEs. RPNs prepared with Alcalase (RPNs-alc) exhibited superior characteristics, including the smallest particle size (≈379.6 nm), a uniform unimodal distribution, the highest content of hydrophobic and antioxidant-related amino acids, the largest three-phase contact angle, and the strongest DPPH radical scavenging activity. These properties enabled RPNs-alc to form HIPEs with a highly ordered, continuous network microstructure, which demonstrated exceptional physical stability, maintaining structural integrity without phase separation over 14 days of storage. In contrast, RPNs derived from Protamex (RPNs-pro) showed larger, polydisperse particles, leading to HIPEs with heterogeneous droplet sizes, interfacial aggregates, and poor stability, resulting in rapid phase separation. RPNs from Trypsin (RPNs-typ) displayed an intermediate performance. The stability of the HIPEs was governed by hydrophobic interactions and hydrogen bonds, the main forces maintaining the RPN structure. The findings demonstrate that enzyme selection critically determines the functionality of RPNs, with Alcalase producing optimal bifunctional stabilizers for fabricating stable, antioxidant-rich Pickering emulsions. These findings establish a clear relationship between enzyme selection, interfacial properties, and emulsion stabilization capacity, providing valuable insights for designing efficient particle stabilizers for food and pharmaceutical applications.

## Figures and Tables

**Table 1:** Amino acid composition of RPNs obtained from Alcalase, Trypsin, and Protamex.

Amino Acid	RP	RPNs-alc	RPNs-typ	RPN-pro
asp	9.62 ± 0.17 ^a^	10.64 ± 0.12 ^c^	10.72 ± 0.1 ^c^	10.03 ± 0.12 ^b^
glu	18.45 ± 0.21 ^d^	17.27 ± 0.54 ^c^	16.54 ± 0.28 ^b^	14.98 ± 0.25 ^a^
ser	4.25 ± 0.03 ^c^	3.34 ± 0.11 ^a^	3.94 ± 0.05 ^b^	4.29 ± 0.06 ^c^
his	2.54 ± 0.08 ^a^	2.78 ± 0.09 ^b^	2.94 ± 0.1 ^c^	2.7 ± 0.09 ^b^
gly	3.68 ± 0.07 ^a^	4.34 ± 0.04 ^b^	4.46 ± 0.07 ^b^	5.68 ± 0.02 ^c^
thr	4.31 ± 0.03 ^d^	2.9 ± 0.04 ^a^	3.03 ± 0.04 ^b^	3.31 ± 0.1 ^c^
arg	9.29 ± 0.05 ^c^	9.42 ± 0.05 ^d^	8.98 ± 0.08 ^b^	7.76 ± 0.08 ^a^
tyr	4.75 ± 0.06 ^c^	3.11 ± 0.01 ^a^	3.79 ± 0.09 ^b^	5.06 ± 0.07 ^d^
ala	5.46 ± 0.04 ^b^	5.5 ± 0.09 ^b^	5.5 ± 0.07 ^b^	5.14 ± 0.04 ^a^
cys-s	0.39 ± 0.1 ^a^	1.88 ± 0.08 ^c^	1.32 ± 0.09 ^b^	0.58 ± 0.08 ^a^
val	6.69 ± 0.02 ^a^	8.91 ± 0.02 ^b^	8.79 ± 0.05 ^b^	8.53 ± 0.07 ^b^
met	1.89 ± 0.07 ^c^	1.07 ± 0.07 ^a^	1.62 ± 0.1 ^b^	0.98 ± 0.12 ^a^
phe	5.62 ± 0.02 ^a^	6.61 ± 0.06 ^c^	6.5 ± 0.04 ^c^	6.03 ± 0.01 ^b^
ile	4.81 ± 0.01 ^a^	5.59 ± 0.12 ^b^	5.12 ± 0.07 ^b^	6.33 ± 0.01 ^c^
leu	8.06 ± 0.08 ^a^	10.92 ± 0.11 ^c^	9.98 ± 0.04 ^b^	9.16 ± 0.04 ^b^
lys	3.62 ± 0.05 ^b^	3.34 ± 0.08 ^a^	3.39 ± 0.07 ^a^	5.62 ± 0.04 ^c^
pro	6.56 ± 0.02 ^c^	4.27 ± 0.02 ^a^	4.09 ± 0.05 ^a^	4.83 ± 0.04 ^b^
THAA	32.93 ± 0.61 ^a^	40.48 ± 0.78 ^d^	38.83 ± 0. 72 ^c^	36.75 ± 0.69 ^b^
TNEAA/TEAA	60.13 ± 0.88 ^a^	70.49 ± 0.79 ^b^	69.74 ± 0.89 ^b^	73.11 ± 0.92 ^c^
TAAA	64.29 ± 0.85 ^b^	68.04 ± 0.93 ^c^	65.6 ± 0.97 ^b^	61.32 ± 0.82 ^a^

THAA—total hydrophobic amino acids, including ala, cys-s, val, met, phe, ile, and leu. TNEAA/TEAA—total non-essential amino acid/total essential amino acid. TAAA—total antioxidant amino acids, including acid, asp, glu, arg, val, phe, leu, and pro. The lowercase letters in the same row indicate a significant difference (*p* < 0.05).

## Data Availability

The original contributions presented in this study are included in the article. Further inquiries can be directed to the corresponding author.
